# Antitumor and Phytochemical Properties of *Ferula assa-foetida* L. Oleo-Gum–Resin against HT-29 Colorectal Cancer Cells In Vitro and in a Xenograft Mouse Model

**DOI:** 10.3390/molecules28248012

**Published:** 2023-12-08

**Authors:** Naglaa Elarabany, Abeer Hamad, Nurah M. Alzamel

**Affiliations:** 1 Department of Biology, College of Science and Humanities, Shaqra University, Shaqra 11961, Saudi Arabia; 2 Zoology Department, Faculty of Science, Damietta University, New Damietta 34517, Egypt; 3 Biology Department, College of Applied and Industrial Science, Bahri University, Khartoum 1660, Sudan

**Keywords:** *Ferula assa-foetida* L., oleo-gum–resin (OGR), ethanolic extract, cytotoxicity, apoptosis, colorectal cancer, HT-29 cell line, xenograft

## Abstract

Colorectal cancer (CRC) is one of the most frequently occurring tumors. *Ferula assa-foetida* oleo-gum–resin (OGR) extract is a traditional cooking spice known for its broad spectrum of biological activities such as antifungal, antiparasitic, and anti-inflammatory activities. This study evaluated the antitumor effect of OGR extract against HT-29 colorectal cancer cells. The OGR chemical composition was analyzed using LC–ESI–MS/MS; MTT, clonogenic assays, and a xenograft model were used to measure cytotoxicity, while apoptotic proteins were detected using Western blotting. Phytochemical analysis revealed that the extract was a rich source of isoflavones, xanthones, and other derivatives. In a dose-dependent manner, the OGR extract significantly inhibited colony formation ability and HT-29 cell growth (IC_50_ was 3.60 ± 0.02 and 10.5 ± 0.1 mg/mL, respectively). On the other hand, the OGR extract significantly induced apoptosis and increased the expression of some pro-death proteins involved in cellular apoptosis including PUMA, BIM, BIK, and BAK. Moreover, in a subcutaneous HT-29 xenograft model, the tumor volume and burden decreased after treatment with the OGR extract (550 ± 32 mm^3^ and 16.3 ± 3.6, respectively) This study demonstrated that *Ferula assa-foetida* OGR ethanolic extract has potential antitumor effects against HT-29 CRC cell lines by reducing cell viability and the function of apoptosis. More studies are needed to reveal the underlying mechanisms related to cytotoxicity and apoptosis induction.

## 1. Introduction

Despite significant advancements and the identification of new anticancer therapies, cancer is considered to be one of the leading causes of mortality worldwide and is responsible for a significant number of fatalities annually [1,2]. Every year, the Food and Drug Administration (FDA) approves new therapies for cancer treatments, but because of severe side effects and different drug resistance patterns, present treatments are no longer suitable; therefore, significant research is needed to find a new agent with less hazardous effects [2,3].

Colorectal cancer (CRC) is one of the most frequent cancers [4] and is responsible for highest mortality rate in developing countries in both sexes [5]. Despite CRC’s current therapy regimens such as chemotherapy, radiation therapy, and surgery, these regimens have several limitations, e.g., cost, drug resistance, intra-tumor heterogeneity, tumor evolution, and other adverse effects [6]. Currently, new antitumor treatments like organ transplantation, targeted therapy, and immunotherapy [7] have been drawing attention as CRC treatment options; however, these too have adverse effects, leading to early mortality risk and drug discontinuation [8,9]. Despite all these efforts, CRC incidence is steadily increasing [4], so new cost-effective treatments with minimal adverse effects are urgently needed. To achieve this, dietary supplements extracted from plants are starting to be recognized as the most effective way to reduce the burden of death associated with colorectal cancer [10].

Medicinal plants are the main source of drug discovery for disease management, including antitumor chemotherapeutic drugs [11,12]. Phytochemicals are natural compounds present in a wide variety of foods and medicinal plants; they are very important in the management and prevention of several diseases. The application of phytochemicals is growing in Western nations due to their anti-inflammatory, antioxidant, and anticancer properties [13].

For CRC, several medicinal plants have proven to have antitumor effects, such as *Zingiber officinale* [10], *Musa* spp. [14], *Hibiscus sabdariffa* [15], *Aloe vera*, and *Aloe arborescens* extracts [16]

In the Middle East and Asia, more than 85% of the population uses herbal medicine for disease management [11]. *Ferula assa-foetida* L. belongs to the family Apiaceace and is an important Ferula species used in traditional medicine for some reproductive system diseases, nervous problems, digestive diseases, and some tumors [17]. The main plant bioactive compound is oleo-gum–resin (OGR), which is extracted from the roots and stems [18]. The resin is usually collected before the plant flowers, and an incision is made in the stem close to the crown and at the upper part of the living rhizome root. A milky juice appears at those cuts. After some days, the juice solidifies and is scraped off; then, a new slice of the root is cut where more latex appears. The collection of resin and the slicing of the root are repeated until exudation stops [19]. Its extracts contain major antioxidant compounds including phenolic compounds like diterpenes, phenolic acids (cinnamic and benzoic acids derivatives), tocopherols, flavonoids, and carotenoids [20]. Assa-foetida OGR is traditionally used as an analgesic, sexual aphrodisiac, anticonvulsant, and anthelmintic agent [17]. In various tumors, the cytotoxic and anticancer effects of *Ferula assa-foetida* L. have been studied [17,21]. 

There is limited information and studies available regarding the antitumor effect of biocompatible *Ferula assa-foetida* L. extract against colon cancer cell lines [22]. Thus, this study aimed to assess the antitumor effects of *Ferula assa-foetida* L. OGR ethanolic extract against HT-29 CRC lines and to identify the optimal concentration that leads to significant tumor cytotoxicity and cellular apoptosis. In addition, this study aimed to assess its safety and effectiveness against tumor volume and burden in a xenograft mouse model.

## 2. Results

### 2.1. Phytochemical Analysis of Ferula assa-foetida L. OGR Extract

**Figure 1:**
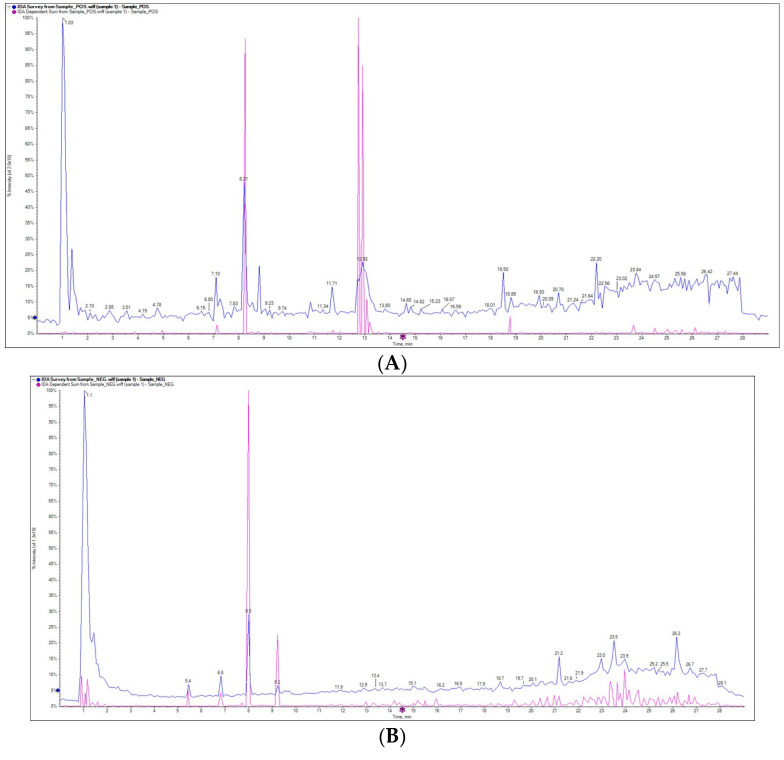
Compounds identified from ethanolic extract of *Ferula assa-foetida* OGR by LC–ESI–MS/MS ((**A**): positive ion mode and (**B**): negative ion mode).

The phytochemicals in *Ferula assa-foetida* L. OGR extract were analyzed by LC–ESI–MS/MS. Figure 1 shows the peak spectra and their mass peaks obtained from the OGR extract. All compounds were identified by interpreting their obtained MS/MS mass spectra. The identified compounds are a rich source of isoflavones, flavonoid glycosides, xanthones, alkaloids, and other derivatives (Table 1). Numerous components were identified in the OGR extract, the highest levels were 10-methylacridone (6.03%), metformin (5.92%), 5-hydroxymethylcytosine (5.88%), three-b-hydroxyaspartate (5.44%), deltamine (5.30%), epicatechin gallate (4.54%), (Z)-2-(3-hydroxy-4-methoxybenzylidene)-9-(quinolin-4-yl)-8,9-dihydro-2H-furo[2,3-f]chromene-3,7-dione (4.49%), taurine (4.47%), indole-3-ethanol (3.83%), and shikimate (3.54%).

### 2.2. Ferula assa-foetida L. OGR Extract Inhibited the Viability of HT-29 CRC Cells and Colony Formation

**Figure 2:**
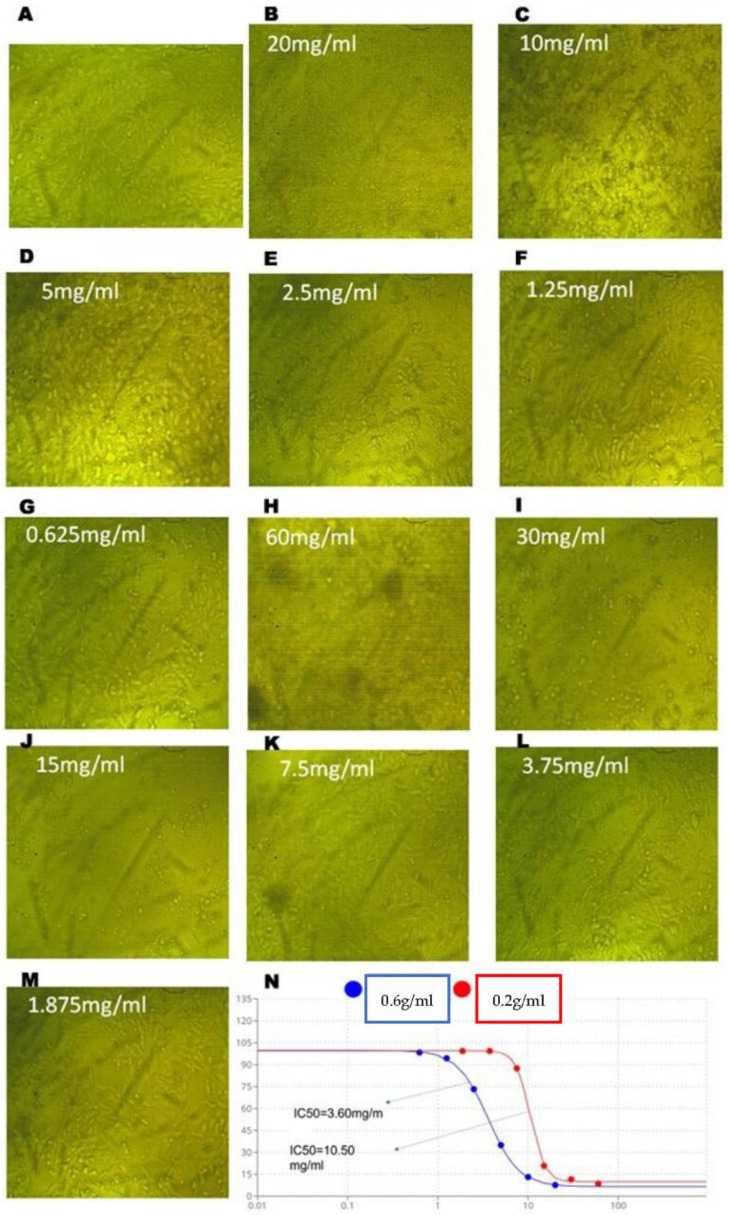
*Ferula assa-foetida* OGR ethanolic extract significantly inhibited the viability of HT-29 cells. (**A**) The control HT-29 cells: the extract exhibited significant cytotoxicity in a dose-dependent manner using the high ((**B**–**G**); 0.6 g/mL) and the low ((**H**–**M**); 0.2g/mL) concentrations. (**N**) The high concentration (IC_50_ 3.60 ± 0.02 mg/mL) was more potent in inhibiting cell proliferation than the low concentration (IC_50_ 10.5 ± 0.1 mg/mL).

**Figure 3:**
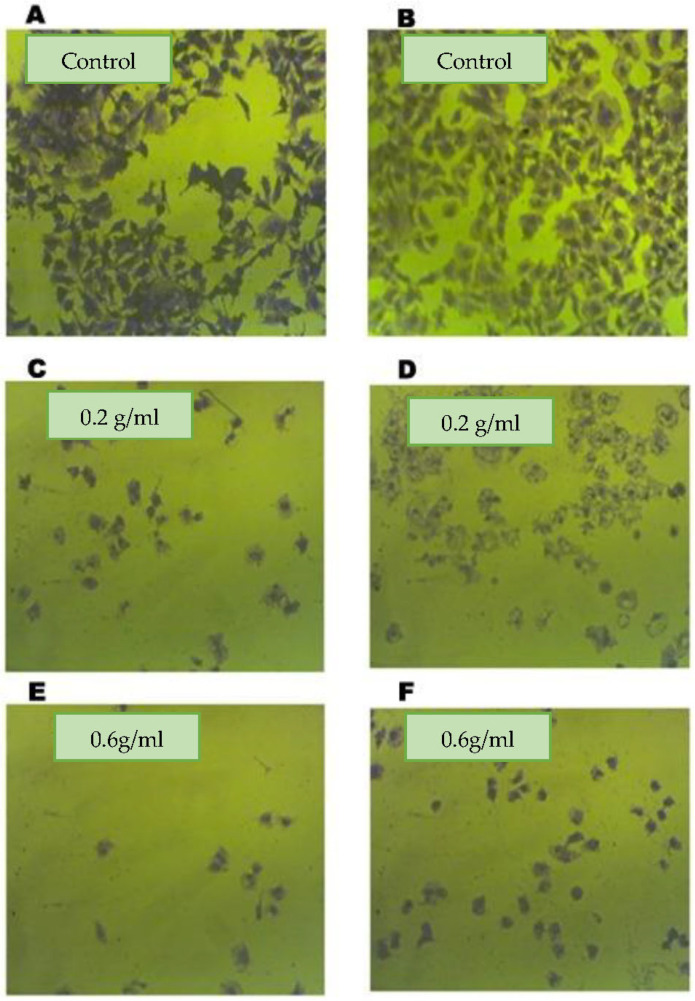
Clonogenic growth ability of HT-29 CRC cells. (**A**,**B**) The control cells; cells treated with both the low ((**C**,**D**); 0.2 g/mL) and the high ((**E**,**F**); 0.6 g/mL) concentrations of *Ferula assa-foetida* OGR ethanolic extract exhibited significant growth inhibition. In four independent experiments, similar results were obtained.

To assess the effects of assa-foetida OGR extract on CRC cell proliferation, HT-29 cells were treated with OGR extract at the indicated concentrations (0.2 g and 0.6 g/mL) for 24 h. As is shown in Table 2 and Figure 2, the assa-foetida OGR extract inhibited cell growth in a dose-dependent manner and the high concentration (0.6 g/mL; IC_50_ 3.60 ± 0.02 mg/mL) was more potent in inhibiting CRC cell proliferation than the low concentration (0.2 g/mL; IC_50_ 10.5 ± 0.1 mg/mL). To further validate the antiproliferative effect of assa-foetida OGR extract, a clonogenic assay was performed on CRC cells. The colony formation ability of HT-29 CRC cells was significantly inhibited in a dose-dependent manner (Figure 3).

### 2.3. Effect of Assa-foetida OGR Extract on Apoptosis and Pro-Death Proteins of CRC Cells

The expression of the BCL-2 family of pre-death proteins involved in cellular apoptosis was examined by Western blotting in HT-29 CRC cells. The relative densities of these proteins, which are central regulators of apoptosis, were increased in a dose-dependent manner.

**Figure 4:**
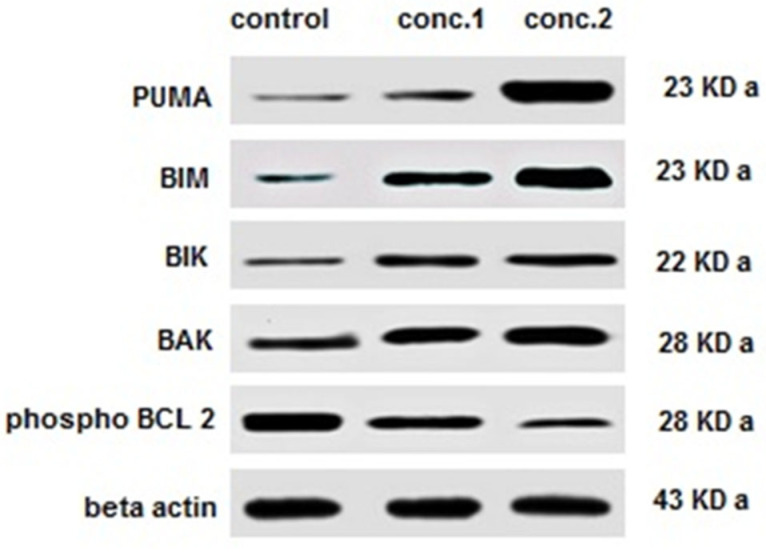
The BCL-2 family of pre-death proteins were detected by Western blotting in CRC cells. The relative densities of these proteins, which are central regulators of apoptosis, were increased in a dose-dependent manner. Similar findings were obtained in three replicates.

In cells treated with assa-foetida OGR extract, BCL-2 family proteins including PUMA, BIM, BIK, and BAK were increased by the OGR ethanolic extract in HT-29 CRC cells after treatment for 24 h. Despite that, phospho-BCL-2 was decreased using assa-foetida OGR extract. The more profound changes were associated with the high concentration of the extract (Figure 4).

### 2.4. Effect of Assa-foetida OGR Extract on a HT-29 Tumor in the Xenograft Model

**Figure 5:**
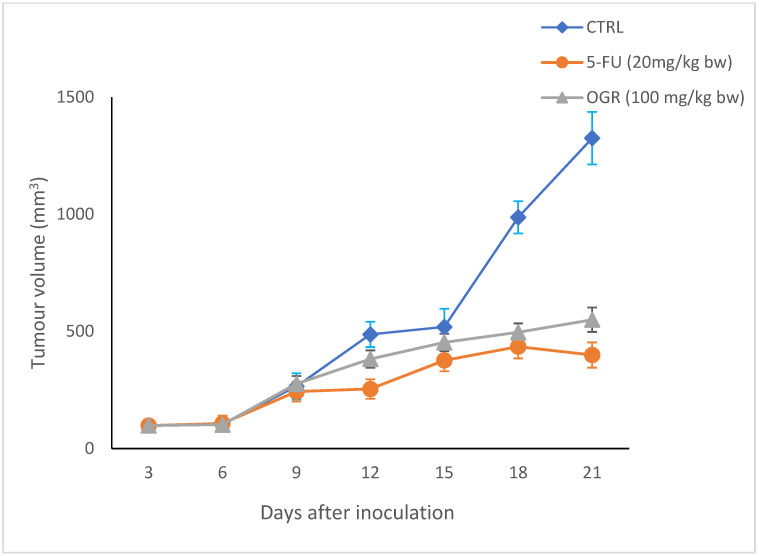
Effect of assa-foetida OGR (100 mg kg bw) on tumor volume in BALB/c nude mice compared to control and 5-FU (20 mg/kg bw) groups.

**Figure 6:**
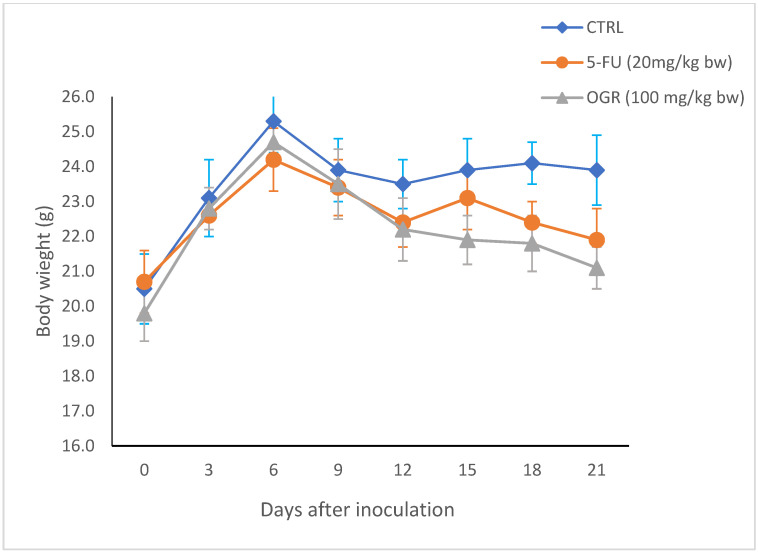
Effect of assa-foetida OGR (100 mg/kg bw) on body weight of BALB/c nude mice compared to control and 5-FU (20 mg/kg bw) groups.

By the end of the experiment, tumors were removed from each mouse for the assessment of tumor weight and evaluation of the antitumor effect of assa-foetida. As is shown in Table 3, there was a significant decrease in tumor weight, tumor volume, and tumor burden in both the OGR-treated group and the 5-FU group as compared to the negative control. In particular, the tumor volume (Figure 5) decreased significantly in both the OGR-treated group and the 5-FU-treated group when compared to the control group (mean ± SD = 550 ± 32, 399 ± 24 and 1325 ± 112, respectively) while the body weight showed no significant changes in all groups. This suggests that assa-foetida OGR administration is safe and has no adverse effects at the chosen dose (Figure 6).

## 3. Discussion

Various chemical compounds have been isolated from different plants. Phytocompounds have become of considerable interest because of their different applications and because of their biological activities and beneficial effects.

Assa-foetida is an OGR obtained from the *Ferula assa-foetida* L. root that is used traditionally as an analgesic, sexual aphrodisiac, anticonvulsant, and anthelmintic agent [17]. New studies have reported that this *Ferula assa-foetida* L. OGR has relaxant, antiviral, anticonvulsant, antidementia, antimutagenic, anti-inflammatory, antidiabetic, antifungal, and particularly, antitumor activities [17]. Its antitumor potential is owing to the identified main compounds including sulfide and glycoside compounds, coumarin derivatives, and various terpenoids [23]. Limited data are available regarding the antitumor effect of *Ferula assa-foetida* L. OGR extract against CRC [22]. At this point, this study aimed to assess the antitumor effects of OGR ethanolic extract against HT-29 CRC including tumor cytotoxicity, antiproliferative effects, and cellular apoptosis.

In this study, the phytochemical assa-foetida OGR ethanolic extract was evaluated and LC–ESI–MS/MS revealed that the extract is a rich source of isoflavones, flavonoid glycosides, xanthones, coumarin, and other derivatives. The chemical composition of the ethanolic OGR extract included taurine as a natural antioxidant [24]; flavonoids such as epicatechin gallate as anti-inflammatory and growth suppressor agents [25]; (Z)-2-(3-hydroxy-4-methoxybenzylidene)-9-(quinolin-4-yl)-8,9-dihydro-2H-furo[2,3-f]chromene-3,7-dione as anticancer, antibacterial, and antiviral compounds [26]; acridone alkaloids such as 10-methylacridone with antitumor, antibacterial, antiviral activities [27]; and diterpenoid alkaloids such as deltamine as analgesic factors [28]. The resin and gum portions of *Ferula assa-foetida* L. are known to contain many compounds, especially sulfur-containing compounds, that have been shown to have significant tumor chemoprevention abilities, in vivo and in vitro [29,30].

Several studies have revealed potential cancer chemopreventive, antimutagenicity, and antioxidant activities of *Ferula assa-foetida* L. extracts [17]. In this study, assa-foetida OGR ethanolic extract (0.2 g and 0.6 g/mL) showed anti-proliferative effects on CRC HT-29-treated cells. Assa-foetida OGR extract, in a dose-dependent manner, inhibited cell growth and the high concentration (IC_50_ 3.60 ± 0.02 mg/mL) was more potent in inhibiting CRC cell proliferation than the low concentration (IC_50_ 10.5 ± 0.1 mg/mL). Furthermore, the colony formation ability of HT-29 CRC cells was significantly inhibited in a dose-dependent manner using assa-foetida OGR extract.

In a similar in vitro study, Abroudi et al. evaluated the anti-proliferative effect of the ethanolic extract of *Ferula assa-foetida* L. on pheochromocytoma (PC12) and breast cancer (MCF7). They reported that the ethanolic extract had a significant effect on apoptosis induction and cell viability [31].

Using general cytotoxicity by artemia salina, Bagheri et al. [32] reported the cytotoxic effect of the Ferula species. Also, tschimgine and stylosin isolated from *Ferula ovina* exhibited cytotoxic activities against the human melanoma cell line (SK-MEL-28) [33]. Additionally, active compounds isolated from *Ferula assa-foetida* L. exerted tumor cytotoxic and antigenotoxic effects against tumor cell lines. As in human peripheral lymphocytes, umbelliprenin showed antigenotoxic properties [34]. In another investigation using an MCF-7 cell line, it was found that prenylated coumarin isolated from Ferula showed cytotoxic effects (IC_50_ = 59.7 mg/mL) [35].

Moreover, in vivo studies reported the antitumor effect of Ferula species. Animal pre-treatment with assa-foetida acetone extract may lead to the reversal of early carcinogenesis events in animals treated with a known cancer promoter (TPA) [36]. In Sprague Dawley rats, the size and multiplicity of palpable mammary tumors were reduced after assa-foetida treatment [29]. Following assa-foetida supplementation in rats, Mallikarjuna et al. demonstrated multiplicity and size reduction in mammary tumors [37]. Bagheri et al. investigated the antitumor effect of assa-foetida ORG extract in vivo using mouse breast carcinoma 4T1 cells [38]. In treated mice, they showed that treatment with ORG extract was effective in reducing cancer weight and volume, and increasing necrotic areas in the cancer tissue [38].

Another interesting result of this study is the effect of assa-foetida OGR extract on cellular apoptosis and pro-death protein expression. In HT-29 cells treated with OGR extract, apoptosis-related proteins including PUMA, BIM, BIK and BAK were increased, while phospho-BCL-2 was decreased. Recent studies have reported that *Ferula assa-foetida* L. essential oil nano-emulsion can lead to apoptosis by decreasing BCL-2 and increasing BAX expression in MCF7 breast cancer cells [21]. Also, using *Ferula assa-foetida* L. extract zinc nanoparticles, another study revealed apoptotic and antioxidant properties accompanied by the upregulation of Bax and downregulation of BCL-2 in HT-29, MDA-MB231, and MCF7 cell lines [39].

## 4. Materials and Methods

### 4.1. Plant Extract Preparation

*Ferula assa-foetida* L. OGR was purchased from a local market; the resin was identified and confirmed by one of the authors (A. H.) and compared to the published plant description [40]. Initially, to prepare the ethanolic extract, 50 g and 150 g of dried OGR was crushed to a fine powder. Separately, each powder weight was soaked for 48 h in 500 mL of 95% ethanol at room temperature. To avoid volatile content preservation, the temperature was raised to 50 °C using a hotplate and kept warm for 5 h. After cooling, the obtained crude ethanol extract was filtered in a vacuum using Whatman filter paper (grade 40) to half its volume to obtain the solutes at 0.2 g/mL and 0.6 g/mL. The solutions were then evaporated and the solid residues were re-suspended in 20% ethanol, and this step was repeated three times. A small portion of the 0.6 g/mL concentration was injected into the LC/MS/MS equipment to identify the active ingredients, and the recovered extracts were stored at 4 °C until the time of the other experiments.

### 4.2. Liquid Chromatography–Electrospray Ionization–Tandem Mass Spectrometry (LC–ESI–MS/MS) Analysis of the Extract

The analysis of the extract was caried out using LC–ESI–MS/MS with an ExionLC AC system for separation and a SCIEX Triple Quad 5500 + MS/MS system supported with electrospray ionization (ESI) for identification. The LC separation was performed using an Ascentis^®^ Express 90 Å C18 Column (2.1 × 150 mm, 2.7 µm) (Sigma-Aldrich, St. Louis, MO, USA). The injection volume was 5 µL, the flow rate was 0.3 mL/min, and the mobile phase was made up of acetonitrile and 5 mM ammonium formate at pH 3. After 50 min of scanning, the mass spectra were obtained from 100 to 1000. The positive electrospray ionization (ESI) mode was applied with the following settings: curtain gas, 25 psi; IonSpray voltage, 5500 for POS and −4500 for NEG; source temperature, 500 °C; ion source gases 1 and 2 were 45 psi and from 50 to 1000 Da for MS2 with a declustering potential, 80 (POS) and −80 (NEG); collision energy, 35 (POS) and −35 (NEG); and collision energy spread, 15. Mass feature extraction of the resulting LC–MS data and maximum peak detection was carried out using the MZmine analysis software package, version 2.3. The identification of compounds was carried out using MS-DIAL.

### 4.3. Cell Culture and Cytotoxicity against HT-29 CRC Tumor Cells

This assay was performed according to Freshney [41]. HT-29 (human colon adenocarcinoma; American Type Culture Collection) cell lines were obtained from the National Cancer Institute (NCI, Egypt) and cultured in RPMI (Roswell Park Memorial Institute)-1640 media containing 2% fetal bovine serum (FBS), and streptomycin and penicillin antibiotics; it was then incubated at 37 °C for 24 h with 5% CO_2_. To evaluate its anticancer efficacy, the MTT (3-(4,5-dimethylthiazol-2-yl)-2,5-diphenyltetrazolium bromide) assay was performed for the OGR extract. CRC cells (105 CFU/mL) were cultured into RPMI medium in 96-well plates and two-fold concentrations of the OGR extracts from the 0.6 g/mL (20 mg/mL, 10 mg/mL, 5 mg/mL, 2.5 mg/mL, 1.25 mg/mL, and 0.625 mg/mL) and 0.2 g/mL (60 mg/mL, 30 mg/mL, 15 mg/mL, 7.5 mg/mL, 3.75 mg/mL, and 1.875 mg/mL) concentrations were subjected to the wells and incubated at 37 °C for 24, 48, and 72 h. The wells were emptied and 20 µL of MTT dye (5 mg/mL in PBS; BIO BASIC CANADA INC., Markham, ON, Canada) with 200 µL of RPMI medium were added and incubated for four hours. The supernatant was discarded and 200 µL of dimethyl sulfoxide (DMSO) was added. Optical density was read at 560 nm and subtracted from the background at 620 nm. The viability of cells was measured using the following formula: viability (%) = OD samples [treated cell lines]/OD controls [untreated cell lines] × 100. Using the Bliss method [42], the half-maximal inhibitory concentration (IC_50_) was obtained. Moreover, using light microscopy, cell cytotoxicity was examined.

### 4.4. In Vitro Clonogenic Assay

Harvesting HT-29 cells was performed using trypsinization the overlying medium was removed and cells were washed with PBS. Then, PBS was removed and replaced by a solution containing trypsin. At a density of 400 cells per well, HT-29 cells were seeded in a 6-well plate. After 24 h and cell attachment, the cells were treated with the solutes (0.2 g/mL and 0.6 g/250 mL). To assess the colony formation rate, colonies were fixed with glutaraldehyde (6% *v*/*v*) or 10% formalin and stained with crystal violet (0.5% *w*/*v*). Using a stereomicroscope, typical images were captured and colonies were counted. The colony formation rate (%) = amount of colonies/number of plated cells × 100.

### 4.5. Immunoblotting

As described previously [43], Western blotting was performed. The total protein of cell lysates was extracted, and the protein concentration was obtained using a bicinchoninic acid (BCA) protein assay reagent kit (Pierce, Rockford, IL, USA). After SDS-PAGE separation, proteins were transferred onto polyvinylidene fluoride (PVDF) membranes (Immobilon, Millipore, Burlington, MA, USA) and then blocked with non-fat milk (5% in PBS–Tween20). Proteins were immunoprecipitated overnight at 4 °C with anti-PUMA (Cell Signaling, Danvers, MA, USA), anti-BIM (Calbiochem, Billerica, MA, USA), anti-BIK (Cell Signaling), anti-BAK (Santa Cruz Biotechnology, Dallas, TX, USA), and anti-Bcl-2 ( Santa Cruz Biotechnology). Precipitates were detected using a 50% slurry of protein G-Sepharose in lysis buffer (GE Healthcare, Amersham, UK), kept overnight at 4 °C. As a loading control, β-actin was used.

### 4.6. Animals and Ethics

After evaluating the antiproliferative effects of assa-foetida in vitro in a dose-dependent manner, and to compare its effect to a commercially available drug (5-FU), a single dose was used in the xenograft model, as previously described [38]. Thirty BALB/c nude mice (male, 4–6 weeks old and body weight 20.7 ± 0.8 g) were obtained from the Uresearch animal facility (URAF), Cairo University, Egypt. The mice were provided with food and water ad libitum and were maintained under pathogen-free conditions. After acclimatization for one week, two million HT-29 cells were subcutaneously injected into the left flank of each mouse (day 0). After the primary tumors had reached a mean volume of approximately 50 mm^3^, the mice were then randomly divided into three groups (*n* = 10); the 1st group was treated with assa-foetida OGR (100 gm/kg body weight) for 21 days post-inoculation; the 2nd group was treated with 5-fluorouracil (5-FU) (20 mg/kg) intraperitoneally, two times a week, considered as a positive control; the 3rd group was used as a negative control. The body weight and tumor size of each mouse were recorded every 3 days. By the end of the experiment, all mice were sacrificed under the effect of diethyl-ether anaesthesia, and tumor sizes (tumor weight, tumor volume, and tumor burden) were calculated according to the following formulae: tumor volume (mm^3^) = [(width)2 × length]/2 and tumor burden (%) = tumor volume (mm^3^)/body weight (g) × 100 [44]. All procedures for anaesthesia/euthanasia and animal experiments were performed following the Institutional Animal Care and Use Committee (IACUC) guidelines. Ethical approval for this study was obtained from the Cairo University Institutional Animal Care and Use Committee (approval number: URAF E-1-23)

### 4.7. Statistical Analysis

Data are expressed as the mean ± SEM. Variables were analyzed by one-way ANOVA with an unpaired *t*-test or Tukey’s test using SPSS (version 20.0; IBM Corp., Armonk, NY, USA); *p* < 0.05 was statistically significant.

## 5. Conclusions

The current study results are very encouraging and showed the antitumor effects of *Ferula assa-foetida* L. OGR ethanolic extract on HT-29 CRC cells. Thus, OGR extract can be considered a medicinal agent in CRC tumor management. It contains many effective compounds that have antitumor effects, which can be used to develop new cancer drugs. These anticancer properties may be related to their great impacts on tumor cytotoxicity and apoptosis initiation. However, further studies are necessary to identify and prove the underlying mechanisms associated with *Ferula assa-foetida* L. OGR extract in inducing tumor cytotoxicity and apoptosis.

## Figures and Tables

**Table 1:** Relevant analytical data of compounds isolated from the assa-foetida OGR extract.

Metabolite Name	RT (min)	*m*/*z*	Type	MS/MS	Level (%)
Taurine	1.00	126.0	Positive	241.1	4.47
5-Aminosalicylic acid	1.03	151.9	Negative	31.5	0.81
Melamine	1.07	127.1	Positive	56.0	1.58
N,n-dimethylaniline	1.13	122.2	Positive	63.2	1.39
2,4,6-Trimethylbenzoic acid	1.03	163.1	Negative	58.4	1.10
1,5-anhydroglucitol	1.03	163.2	Negative	58.2	0.50
2-mercaptobenzothiazole	1.03	165.8	Negative	55.6	0.41
N-acetylglutamate	1.03	187.9	Negative	332.8	2.67
Kynurenic acid	1.03	188.0	Negative	301.2	2.28
Isoquinoline	1.13	130.0	Positive	61.2	0.75
Gluconate	1.03	195.0	Negative	81.6	1.00
Indole-3-carbinol	1.13	130.1	Positive	67.1	0.92
Metformin	1.13	130.2	Positive	305.2	5.92
5-hydroxymethylcytosine	1.11	140.0	Negative	452.0	5.88
Threo-b-hydroxyaspartate	1.11	147.8	Negative	300.4	5.44
Methionine	1.11	148.1	Negative	313.6	2.57
L-histidine	1.11	154.2	Negative	262.6	2.53
Shikimate	1.11	155.0	Negative	906.1	3.54
Menthol(-)	1.11	155.2	Negative	655.3	2.64
10-methylacridone	2.87	210.0	Positive	1102.8	6.03
Indole-3-ethanol	1.11	160.1	Negative	452.4	3.83
Trichloroacetic acid	1.11	160.8	Negative	189.1	1.12
Methionine sulfoxide	1.11	164.0	Negative	162.3	1.02
2-(4-chlorophenyl)-1-(2,4,6-trihydroxyphenyl) ethenone	1.18	277.0	Negative	277.7	0.73
4-aminophenylsulfone	1.27	246.9	Negative	26.7	0.29
2-Aminoethylphosphonic acid	1.43	124.1	Negative	25.5	0.28
S-Adenosyl-L-methionine	7.10	226.9	Positive	53.9	0.38
Brugierol	2.46	136.9	Negative	75.6	1.42
Urocanate	2.46	137.0	Negative	68.4	1.00
Crotetamide	7.10	227.2	Positive	22.8	0.37
Umbelliferone	5.44	161.2	Negative	39.7	1.44
(3as,3a1s,10br,13S)-3a-ethyl-5-(methoxycarbonyl)-1,2,3,3a,3a1,4,6,11,12,13-decahydroindolizino [8,1-cd]carbazol-13-ium iodide	8.28	340.2	Positive	27.6	0.35
N-acetyl-L-cysteine	5.44	162.0	Negative	48.3	0.46
6-hydroxyoctadec-4-enoic acid	8.28	340.3	Positive	18.3	0.40
Sphingomyelin	8.02	713.2	Negative	65.6	0.12
Verminoside	8.02	713.3	Negative	34.3	0.10
Quinovin	8.07	677.4	Negative	41.9	0.27
Ononin	8.81	453.1	Positive	11.3	0.13
Carnosic acid diacetate methyl ester	8.81	453.2	Positive	306.5	2.32
(+)-A-tocopherol	8.81	453.4	Positive	238.3	1.45
4-(4-Decanyl)benzenesulfonic acid	13.29	297.0	Negative	20.4	0.24
2-[4-(Diethylamino)-2-hydroxybenzoyl] benzoic acid	14.19	312.1	Negative	26.2	0.29
Sinomenine	15.45	328.2	Negative	192.4	1.18
Armexifolin	17.39	247.2	Negative	105.5	0.85
Indinavir	21.48	612.2	Negative	60.8	0.28
Oregonoyl A	21.48	623.2	Negative	382.8	1.99
N-Butyryl coenzyme A lithium salt hydrate	22.78	836.7	Negative	42.3	0.34
3-(5-phenylthiophen-2-yl)prop-2-vinyl Acetate	22.97	255.0	Negative	44.4	0.35
Palmitic acid	22.97	255.2	Negative	15.1	0.94
3-cyanopyridine	11.70	105.0	Positive	24.1	1.23
2-methoxy-5-methyl-3-(2-methylbut-3-en-2-yl)chromen-4-one	22.97	257.2	Negative	125.5	0.46
Estrone	23.96	268.8	Negative	162.7	0.51
Homatropine	12.77	276.1	Positive	322.4	0.70
1-(3,4-Dimethoxycinnamoyl) piperidine	12.77	276.2	Positive	317,664.9	0.73
Canrenone	26.17	339.2	Negative	119.9	0.87
Mgmg 18:3	27.22	559.3	Negative	15.5	0.14
(2R)-1-[2-(hydroxymethyl)-5,5,8a-trimethyl-1,4,4a,6,7,8-hexahydronaphthalen-1-yl]-2-methylbut-3-en-2-ol	28.59	236.9	Negative	22.4	0.19
Atropine	13.07	290.2	Positive	6.5	0.02
N-acetylgalactosamine	13.19	244.1	Positive	19.0	0.07
3,3’,4’,7-tetrahydroxyflavylium chloride	13.27	272.0	Positive	29.6	0.25
(E)-8-(1-(hydroxyimino)ethyl)-4,9-dimethyl-2H-furo[2,3-h]chromen-2-one	13.27	272.2	Positive	40.1	0.13
Myristoyl ethanolamide	13.27	272.3	Positive	403.8	2.65
(Z)-2-(3-hydroxy-4-methoxybenzylidene)-9-(quinolin-4-yl)-8,9-dihydro-2H-furo[2,3-f]chromene-3,7-dione	13.89	466.1	Positive	568.3	4.49
Deltamine	13.89	466.2	Positive	474.3	5.30
Epicatechin gallate	14.82	460.1	Positive	779.1	4.54
Apixaban	14.82	460.2	Positive	22.1	0.15
Hesperidin	20.09	645.1	Positive	29.3	0.09
Propanoic acid	21.76	959.4	Positive	75.6	0.91
A-D-Glucopyranoside, β-D-glucopyranosyl	26.35	853.3	Positive	30.1	0.12
Methoxy-mca-albicidin	26.44	857.2	Positive	7.6	0.08

**Table 2:** Effects of assa-foetida OGR ethanolic extract at different concentrations on the viability of HT-29 CRC cells.

Variable	mg/mL	Mean O.D.	SEM	Viability %	Toxicity %	IC_50_ ± SD
HT-29	—	0.779	0.004	100	0	Mg
0.6 g/mL	20	0.059	0.001	7.62	92.38	3.60 ± 0.02 mg/mL
	10	0.102	0.005	13.10	86.91	
	5	0.272	0.006	34.92	65.08	
	2.5	0.570	0.005	73.21	26.79	
	1.25	0.735	0.006	94.35	5.65	
	0.625	0.766	0.007	98.33	1.67	
0.2 g/mL	60	0.066	0.006	8.47	91.53	10.50 ± 0.1 mg/mL
	30	0.09	0.005	11.55	88.45	
	15	0.162	0.005	20.75	79.25	
	7.5	0.681	0.004	87.46	12.54	
	3.75	0.774	0.003	99.32	0.68	
	1.875	0.774	0.003	99.32	0.68	

Mean O.D.: mean optical density; SEM: standard error of the mean; SD: standard deviation.

**Table 3:** Effect of assa-foetida OGR ethanolic extract on tumor volume, tumor weight, and tumor burden in BALB/c nude mice as compared to 5-FU (20 mg/kg bw) and control groups. Values are represented by means ± SD; * indicates significant results.

Groups (*n* = 10)	Tumor Volume (mm^3^)	Tumor Weight (g)	Tumor Burden (%)
Control	1325 ± 112	1.8 ± 0.4	72.6 ± 8.3
5-FU (20 mg/kg bw)	399 ± 24 *	0.7 ± 0.2 *	9.2 ± 1.9 *
Assa-foetida OGR (100 mg/kg bw)	550 ± 32 *	1.2 ± 0.3 *	16.3 ± 3.6 *

## Data Availability

All of the relevant data are presented within the paper.
